# Interfering with reconsolidation by rimonabant results in blockade of heroin-associated memory

**DOI:** 10.3389/fphar.2024.1361838

**Published:** 2024-03-21

**Authors:** Jiang Lin, Yilin Peng, Jinlong Zhang, Junzhe Cheng, Qianqian Chen, Binbin Wang, Yuhang Liu, Shuliang Niu, Jie Yan

**Affiliations:** ^1^ Department of Forensic Science, School of Basic Medical Science, Central South University, Changsha, Hunan, China; ^2^ Department of Forensic Science, School of Basic Medical Science, Xinjiang Medical University, Urumqi, China; ^3^ Clinical Medicine Eight-Year Program, Xiangya School of Medicine, Central South University, Changsha, Hunan, China; ^4^ Department of Anatomy, School of Basic Medical Science, Xinjiang Medical University, Urumqi, China

**Keywords:** reconsolidation, heroin-associated memory, rimonabant, relapse, heroin-seeking

## Abstract

Drug-associated pathological memory remains a critical factor contributing to the persistence of substance use disorder. Pharmacological amnestic manipulation to interfere with drug memory reconsolidation has shown promise for the prevention of relapse. In a rat heroin self-administration model, we examined the impact of rimonabant, a selective cannabinoid receptor indirect agonist, on the reconsolidation process of heroin-associated memory. The study showed that immediately administering rimonabant after conditioned stimuli (CS) exposure reduced the cue- and herion + cue-induced heroin-seeking behavior. The inhibitory effects lasted for a minimum of 28 days. The effect of Rimonabant on reduced drug-seeking was not shown when treated without CS exposure or 6 hours after CS exposure. These results demonstrate a disruptive role of rimonabant on the reconsolidation of heroin-associated memory and the therapeutic potential in relapse control concerning substance use disorder.

## 1 Introduction

Substance use disorder is a persistent, relapsing brain disease driven by several dysfunctional neurobiological and psychological elements including maladaptive learning. (Volkow et al., 2016). Interest has been generated concerning the possibility of weakening or erasing drug-associated memory through blocking memory reconsolidation, a process during which retrieval-dependent synaptic destabilization and ensuing restabilization process occur for subsequent memory expression, thus previously consolidated memory can be updated (Exton-McGuinness and Milton, 2018; Liu et al., 2019).

Accumulating evidence demonstrates that pharmacological intervention targeting reconsolidation results in drug memory disruption and alleviation of drug seeking behavior. For example, U0126 or anisomycin can modify the unstable memory associated with drug use and reduce conditional place preference (CPP) behavior, as well as the seeking of drugs such as morphine and cocaine (Lv et al., 2015; Sorg et al., 2015). Further, an infusion of PKA inhibitors into mice’s amygdala proved to be effective in reducing heroin-seeking (Zhang et al., 2022). Psilocybin significantly decreased alcohol-seeking behavior by interfering with the process of memory reconsolidation (Benvenuti et al., 2023). Additionally, intraperitoneal injection of CB1 receptor antagonist AM251 after memory reactivation modulated cellular and synaptic mechanisms in the basolateral amygdala and reduced subsequent cocaine-seeking behavior (Higginbotham et al., 2021b). However, direct infusion of AM251 into the basolateral amygdala enhanced subsequent cocaine-seeking behavior, indicating an intricate role of the CB1 receptor in terms of substance use disorders (Higginbotham et al., 2021a). Rimonabant, a selective cannabinoid receptor indirect agonist, has been identified to reduce behaviors related to the pursuit of cocaine, nicotine, and alcohol by disrupting the link between drug cues and their rewarding effects in animal models (Le Foll and Goldberg, 2005; Colombo et al., 2007; Butler and Le Foll, 2020). Nevertheless, evidence of its impact on heroin-seeking and relapse remains limited (De Vries et al., 2003; Fattore et al., 2011). Herein, we aimed to assess whether rimonabant blocks the reconsolidation process of heroin-associated memory when administrated at different time intervals and with or without conditioned stimuli (CS) by using a self-administration model.

## 2 Materials and methods

### 2.1 Subjects and drugs

Male SD rats (260–280 g) were used in this experiment. Rimonabant was dissolved in a vehicle solution (Tween 80 in 2.5% dimethyl sulphoxide and 10% cremophor in saline, henceforth referred to as VEH). Four tests with a total of eighty rats were conducted. The VEH group (n = 10) and the rimonabant group (n = 10) each included ten rats apiece. During the experiment, 12 rats were excluded due to catheter failure. The environment in which they were housed had a 12-h reverse light-dark cycle, a constant humidity of around 50%, and a temperature of 23°C ± 2 °C. Water and food were also freely available to them. The Central South University Institutional Animal Care and Use Committee criteria were followed for the experiment procedure.

### 2.2 Intravenous surgery

Rats were given 60 mg/kg of sodium pentobarbital intraperitoneally to produce anesthesia. A catheter was placed into the right jugular vein, ending at the right atrial aperture (Ambroggi et al., 2008; Chen et al., 2021). The catheter was fixed to the cranium of the rat. To prevent intravenous catheter block, 0.1 mL of heparinized saline (Hospira) was given every 2 days. The experiment began five to 7 days after the rats recovered.

### 2.3 Behavioral procedures

#### 2.3.1 Heroin self-administration training

Heroin self-administration training methods remained similar to those of previous studies (Chen et al., 2021; Qian et al., 2022). Two nose-poke devices were set in the operating room, with light stimulation 5 cm above the floor. Rats received 0.05 mg/kg/infusion of heroin intravenously for 10 days, with three one-hour training sessions each day separated by 5 minutes. A fixed-ratio 1 reinforcement regimen was used, accompanied by a 40-s time-out following each infusion. The rats were linked to a tube made of a polyethylene-covered metal cable that went through a fluid rotator and was joined to a 10-mL syringe pump. A 5-s tone-light cue was followed by an intravenous heroin infusion when the rat activated the device with active nose-poke. An inactive nose-poke was also recorded when triggered, but no action was taken.

#### 2.3.2 Nose-poke extinction

24 h after the final heroin self-administration session, a process of ten-day nose-poke extinction was initiated. Within the 3 hours of daily training, there were no consequences for active or inactive nose-pokes (i.e., no intravenous heroin or conditioned tone-light cues). The number of active nose-pokes on two consecutive days less than 20% of the average active nose-pokes on the final 3 days of heroin self-administration was used to determine the extinction effect.

#### 2.3.3 Reactivation of heroin memory

24 h after the nose-poke extinction, a fifteen-minute session was conducted to activate heroin-associated memories (Experiments 1, 2, and 4). The identical retrieval conditions as those for heroin self-administration were used, with the difference that drug signals were used to encourage active nose-pokes instead of heroin.

#### 2.3.4 Cue extinction

The daily three-hour cue extinction was conducted in the same experimental condition as the self-administration training, with the exception that the cue (the tone-light cue) was delivered without a heroin infusion.

#### 2.3.5 Rimonabant treatment

Following the 15-min cue reactivation session, rimonabant or VEH (3 mg/kg, i.p.) was injected. Rats in Experiment 3 also received injections of rimonabant or VEH but were not exposed to CS. In Experiment 4, rats received rimonabant and VEH injections 6 hours after CS exposure. All injections were administered intraperitoneally.

#### 2.3.6 Cue-induced reinstatement of heroin seeking (experiments 1-4)

Cue-induced reinstatement tests were conducted 24 h after rimonabant or VEH administration. The procedure is similar to heroin self-administration, except that the active nose-pokes solely produced tone-light cues and there was no heroin infusion.

#### 2.3.7 Heroin + cue-induced reinstatement (experiments 1, 3, and 4)

Rats were placed in a self-administered training environment after receiving an intraperitoneal injection of 0.25 mg/kg heroin, which was less than the training dose. The condition was similar to heroin self-administration, except that the active nose-pokes only generated tone-light stimuli, and heroin was not infused.

### 2.4 Specific experiments

#### 2.4.1 Experiment 1: the effect of rimonabant on cue-induced and heroin + cue-induced seeking behavior immediately after the retrieval of heroin-associated memory

**FIGURE 1:**
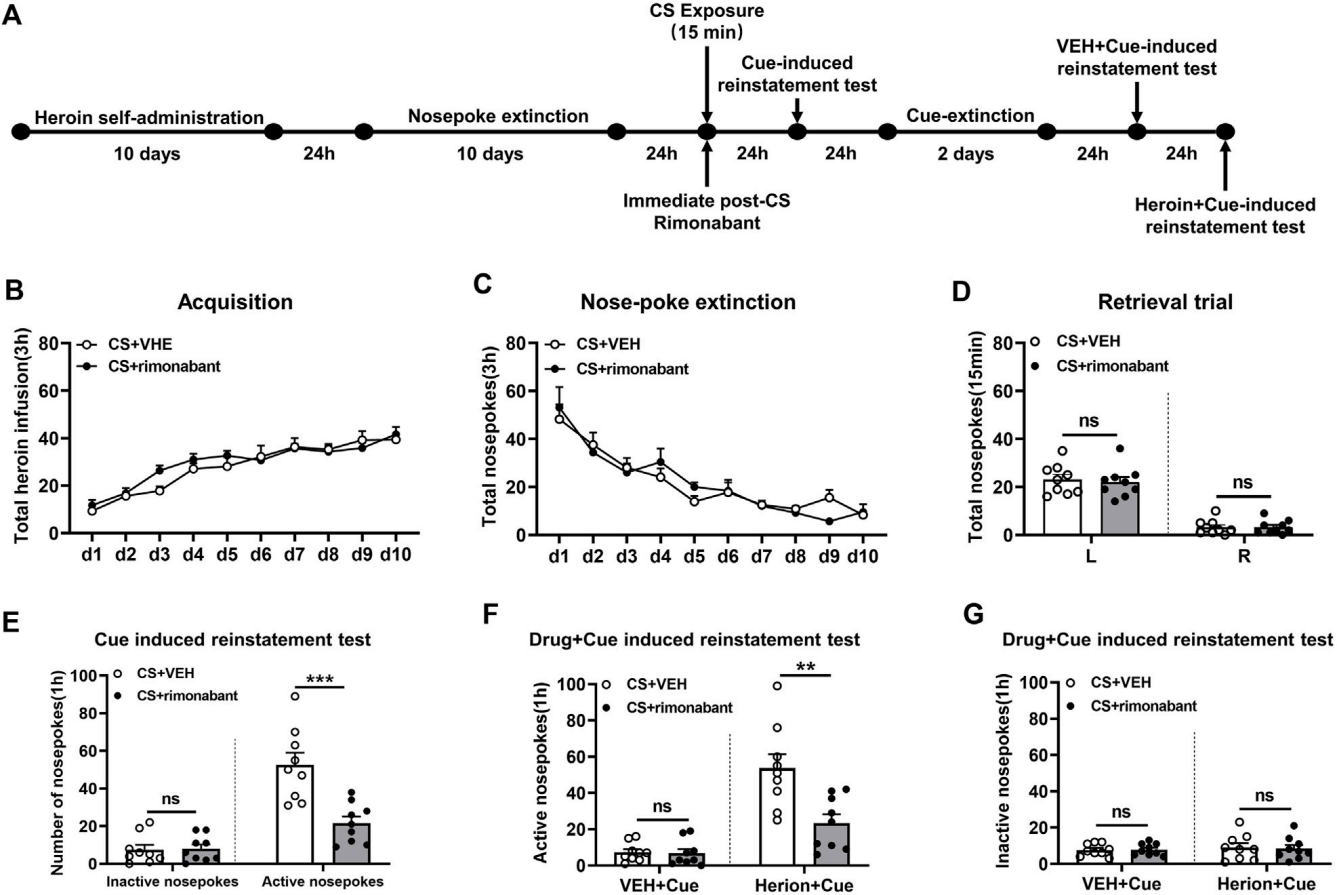
Injection of rimonabant immediately after CS exposure reduced reinstatement of heroin-seeking behaviors induced by heroin-associated cue or heroin + cue priming. **(A)** Experimental flow chart. **(B)** The total number of heroin infusions during heroin self-administration. **(C)** Active nose-poke responses during extinction sessions. **(D)** Nose-poke responses during the reactivation trial. **(E)** Active (left) and inactive (right) nose-poke responses during the cue-induced reinstatement test. **(F,G)** Active (left) and inactive (right) nose-poke responses in VEH or heroin + cue-induced reinstatement test.

Rats received ten consecutive days of self-administration training and subsequent ten consecutive days of nose-poke extinction 24 h later. Then rats were given 15 min of conditioned stimulation in the training context to activate heroin-associated memory 24 h after extinction. Immediately after CS exposure, rats were intraperitoneally injected with rimonabant or an equivalent volume of VEH. Cue-induced reinstatement tests were conducted on the next day and then the rats received 2 days of cue extinction 24 h later. The VEH + cue-induced reinstatement test was conducted on the next day. Then the heroin + cue-induced reinstatement test was performed 24 h later (Figure 1A).

#### 2.4.2 Experiment 2: the effect of rimonabant treatment after memory reactivation on spontaneous recovery of heroin-seeking behavior after 28-day abstinence

**FIGURE 2:**
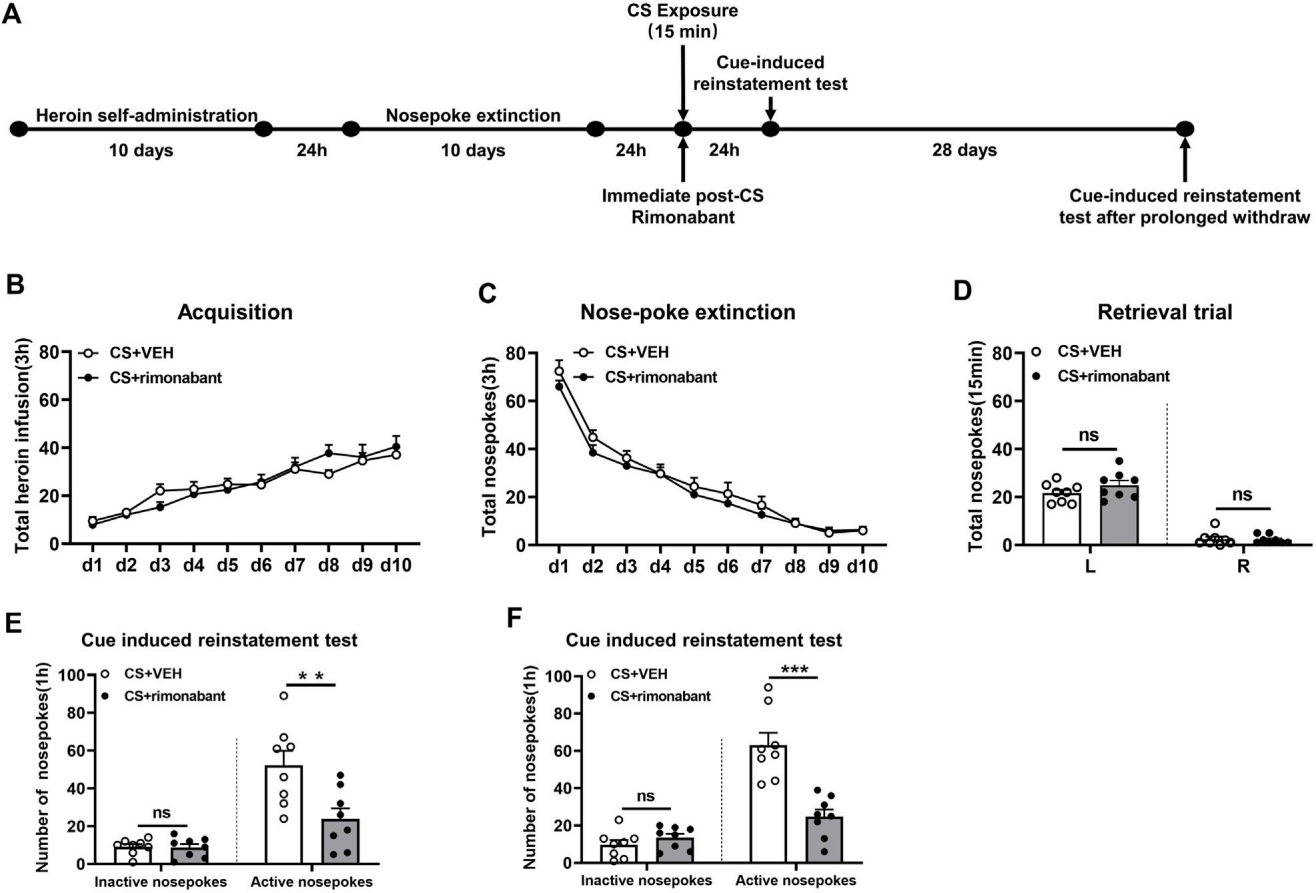
Injecting rimonabant immediately after CS exposure reduced subsequent heroin induction as well as cue-induced recovery of heroin-craving behavior and lasted for at least 28 days. **(A)** Experimental flow chart. **(B)** The total number of heroin infusions during heroin self-administration. **(C)** Active nose-poke responses during extinction sessions. **(D)** Nose-poke responses during the reactivation trial. **(E)** Active (left) and inactive (right) nose-poke responses during the cue-induced reinstatement test. **(F)** Active (left) and inactive (right) nose-poke processes during the cue-induced recovery test after 28 days of withdrawal.

Rats were subjected to self-administration training and 10 days of nose-poke extinction, the same as that of Experiment 1. Following 15 minutes of CS reactivation, rats received intraperitoneal rimonabant administration. After a 24-h interval, the cue-induced reinstatement test was conducted. Thereafter, the test was performed again after a 28-day withdrawal period (Figure 2A).

#### 2.4.3 Experiment 3: effect of rimonabant on cue-induced and heroin + cue-induced seeking behavior without the reactivation of heroin-associated memory

**FIGURE 3:**
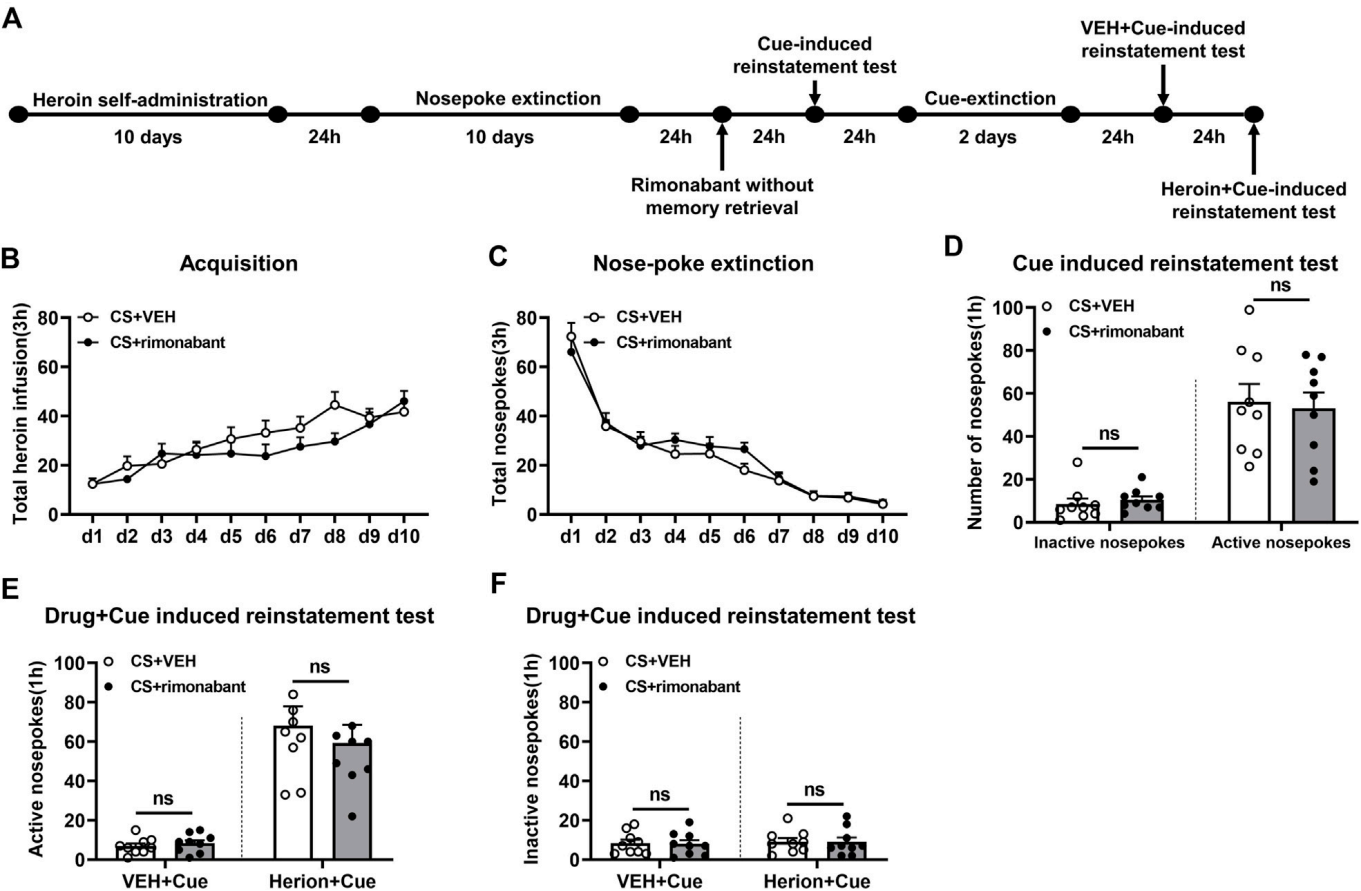
Injection of rimonabant without CS exposure did not affect subsequent cue induction or heroin + cue-initiated heroin-seeking recovery. **(A)** Experimental flow chart. **(B)** The total number of heroin infusions during heroin self-administration. **(C)** Active nose-poke responses during extinction sessions. **(D)** Active (left) and inactive (right) nose-poke responses during the cue-induced reinstatement test. **(E,F)** Active (left) and inactive (right) nose-poke responses in VEH or heroin + cue-induced reinstatement test.

The experimental protocol followed the same steps as those in Experiment 1, except that rats were given an infusion of rimonabant or VEH without memory reactivation (Figure 3A).

#### 2.4.4 Experiment 4: effect of rimonabant on cue-induced seeking behavior and heroin + cue-induced seeking behavior 6 hours after retrieval of heroin-associated memory

**FIGURE 4:**
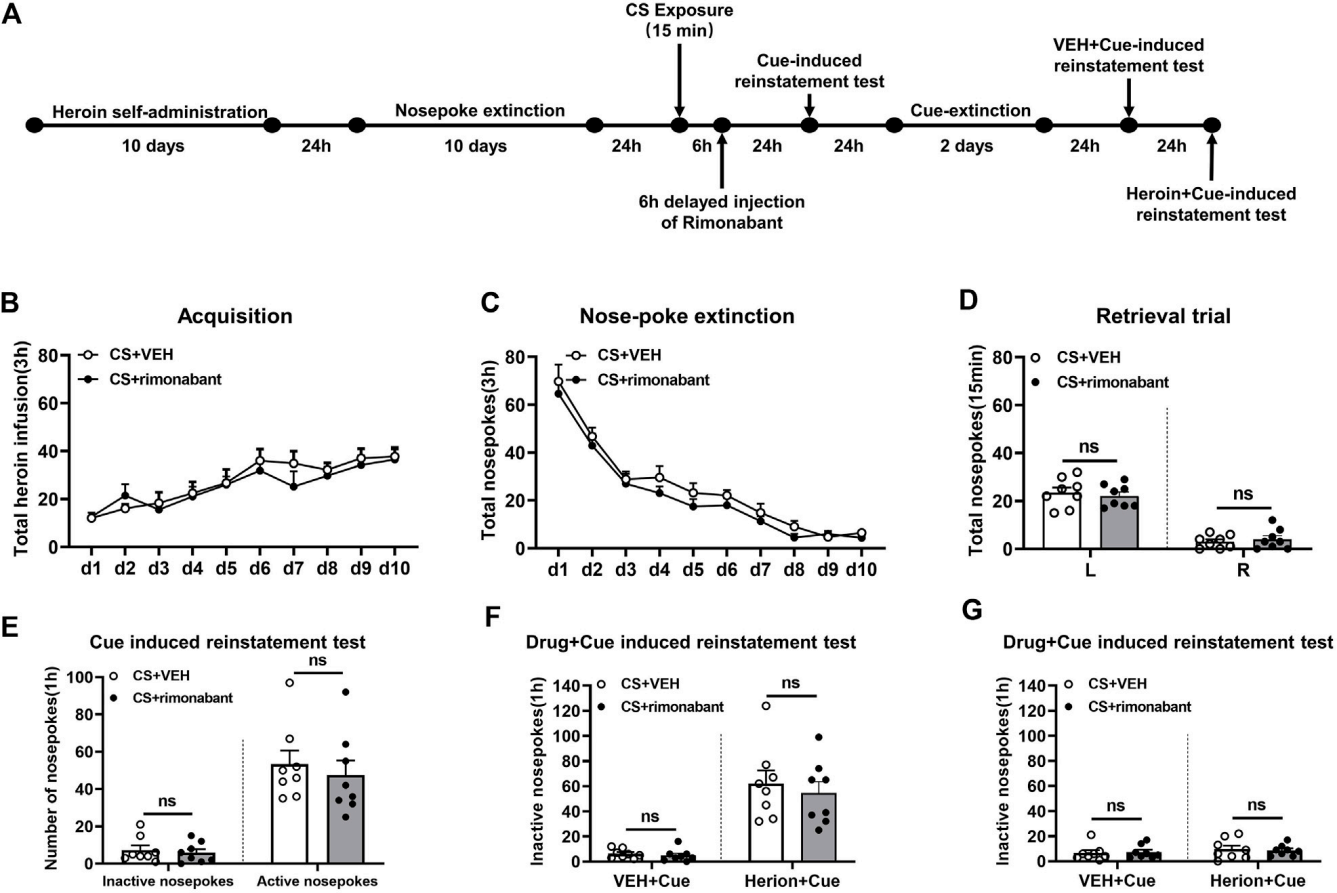
Injection of rimonabant 6 h after CS exposure did not affect subsequent heroin-seeking recovery. **(A)** Experimental flow chart. **(B)** The total number of heroin infusions during heroin self-administration. **(C)** Active nose-poke responses during extinction sessions. **(D)** Nose-poke responses during the reactivation trial. **(E)** Active (left) and inactive (right) nose-poke responses during the cue-induced reinstatement test. **(F,G)** Active (left) and inactive (right) nose-poke responses in VEH or heroin + cue-induced reinstatement test.

The process of Experiment 4 was the same as that of Experiment 1. The difference was that rimonabant was given 6 hours after 15-min CS activation (Chen et al., 2021) (Figure 4A).

### 2.5 Statistical analysis

We employed GraphPad V.8.0 software for data analysis, and the mean ± SEM is the format in which all data results are provided. The analytical method was *t*-test and repeated measures analysis of variance (ANOVA). The test condition was an intra-group factor, while the intervention strategy was an inter-group factor. Tukey’s *post hoc* test was employed concurrently to examine variations among groups. The presence of *p* < 0.05 indicated the existence of a significant difference.

## 3 Results

### 3.1 Experiment 1: blocking the CB1 receptor with rimonabant immediately after conditioned stimulation reduced reinstatement of heroin-seeking behavior

Two groups including the rimonabant group (n = 9) and the VEH group (n = 9) were involved in this procedure. Heroin self-administration training did not result in an appreciable difference in the number of active nose-pokes between the two groups [training day is the main factor: F (9,144) = 36.40, *p* < 0.0001; rimonabant administration is the main factor: F (1,16) = 0.557, *p* = 0.466; interaction between rimonabant administration × training day: F (9,144) = 1.173, *p* = 0.3165, Figure 1B]. No difference was observed between the two groups of active and inactive nose-pokes over ten-day extinction [extinction day is the main factor: F (9, 144) = 28.17, *p* < 0.0001; rimonabant administration is the main factor: F (1,16) = 0.005314, *p* = 0.9428; interaction between rimonabant administration × extinction day: F (9, 144) = 0.9331, *p* = 0.4985, Figure 1C]. The number of active and inactive nose-pokes across the two groups did not differ in the retrieval test [different nose-pokes is the main factor: F (1,16) = 118.5, *p* < 0.0001; rimonabant administration is the main factor: F (1,16) = 0.1348, *p* = 0.7183; interaction of rimonabant administration × different nose-pokes: F (1,16) = 0.09785, *p* = 0.7585, Figure 1D].

The number of active nose-pokes in the test of cue-induced reinstatement varied significantly between the two groups, according to a *t*-test (*p* = 0.0006). Tukey’s *post hoc* analysis demonstrated a significant reduction in heroin-seeking behavior (*p* < 0.0001) (Figure 1E) between the two groups in the cue-induced reinstatement test. In contrast, no difference was found in the number of inactive nose-pokes between the VEH and the rimonabant group (*p* > 0.05) (Figure 1E).

In the heroin + cue-induced reinstatement test, a statistical difference was found in the number of active nose-pokes between the two groups [test condition is the main factor: F (1,16) = 60.01, *p* < 0.0001; rimonabant administration is the main factor: F (1,16) = 8.236, *p* = 0.0111; interaction of rimonabant administration × test condition: F (1,16) = 13.31, *p* = 0.0022, Figure 1F]. When comparing the rimonabant group to the VEH group in the cue-induced reinstatement test, *post hoc* analysis revealed a substantial decrease in heroin-seeking behavior (*p* = 0.0002). The number of inactive nose-pokes, however, did not differ statistically [test condition is the main factor: F (1,16) = 0.3562, *p* = 0.5590; rimonabant administration is the main factor: F (1,16) = 0.01520, *p* = 0.9034; interaction of rimonabant administration × test condition: F (1,16) = 0.07037, *p* = 0.7942, Figure 1G]. According to these results, using rimonabant to block the CB1 receptor immediately following conditioned stimulation reduced the reinstatement of heroin-seeking behaviors.

### 3.2 Experiment 2: blocking the CB1 receptor with rimonabant immediately after CS exposure attenuates heroin-seeking behavior in a long-lasting manner

We aimed to examine how cue-induced reinstatement during extended withdrawal was impacted by rimonabant intervention following CS exposure. Similar to experiment 1, rats were divided into two groups and given different treatments: VEH (n = 8) and rimonabant (n = 8). During heroin self-administration training, no difference was found in the number of active nose-pokes between the two groups [training day is the main factor: F (9,126) = 30.91, *p* < 0.0001; rimonabant administration is the main factor: F (1,14) = 0.006203, *p* = 0.9383; interaction of rimonabant administration × test condition: F (9,126) = 1.328, *p* = 0.2287, Figure 2B]. Two-way repeated ANOVA revealed no statistical difference between the two groups regarding the number of active nose-pokes during the nose-poke extinction [extinction day is the main factor: F (9,126) = 120.8, *p* < 0.0001; rimonabant administration is the main factor: F (1,14) = 1.232, *p* = 0.2857; interaction of rimonabant administration × test condition: F (9,126) = 0.5750, *p* = 0.8156, Figure 2C]. The number of active and inactive nose-pokes in the memory recall test did not differ between the rimonabant intervention group and the VEH control group [different nose-pokes is the main factor: F (1,16) = 210.0, *p* < 0.0001; rimonabant administration is the main factor: F (1,16) = 1.386, *p* = 0.2588; interaction of rimonabant administration × test condition: F (1,16) = 1.476, *p* = 0.2445, Figure 2D].

The number of active nose-pokes between the two groups varied significantly in the cue-induced reinstatement tests which was consistent with the findings of Experiment 1 (*p* = 0.0092) (Figure 2E). The *post hoc* analysis in the cue-induced reinstatement test showed that heroin-seeking behavior was considerably lower in the intervention group in comparison to the VEH group (*p* = 0.0005). The number of inactive nose-pokes did not show a significant difference between the two groups (*p* > 0.05) (Figure 2E). Following 28 days of abstinence, a difference was observed in the number of active nose-pokes in the cue-induced reinstatement test between the two groups (*p* = 0.0002) (Figure 2F). The seeking behavior in the rimonabant group was considerably lower (*p* < 0.0001) than that in the VEH group. Between the two groups, there was no difference in the number of inactive nose-pokes (*p* > 0.05) (Figure 2F). These findings imply that cue- and herion + cue-induced heroin-seeking behavior may be considerably suppressed for at least 28 days by rimonabant administered right after CS exposure.

### 3.3 Experiment 3: intraperitoneal injection of rimonabant to block CB1 receptor without memory reactivation did not attenuate heroin-seeking behavior

This part focuses on whether memory reactivation is a prerequisite for rimonabant-induced impairment of drug-seeking behavior. During heroin self-administration training, no difference was shown in the frequency of active nose-pokes between the VEH group (n = 9) and the rimonabant group (n = 9) [training day is the main factor: F (9,144) = 17.46, *p* < 0.0001; rimonabant administration is the main factor: F (1,16) = 1.115, *p* = 0.3067; interaction of rimonabant administration × training day: F (9,144) = 1.675, *p* = 0.1002, Figure 3B]. Additionally, throughout the nose-poke extinction, no statistical difference was found in the number of active nose-pokes between the two groups [extinction day is the main factor: F (9,144) = 80.52, *p* < 0.0001; rimonabant administration is the main factor: F (1,16) = 0.3931, *p* = 0.5395; interaction of rimonabant administration × extinction day: F (9,144) = 0.8835, *p* = 0.5416, Figure 3C].

In the cue-induced reinstatement test, no statistical difference was revealed between the rimonabant group and the control group concerning active and inactive nose-poke data (*p* > 0.05) (Figure 3D). In the heroin + cue-induced reinstatement test, no discernible difference was found in the number of active nose-pokes [test condition is the main factor: F (1,16) = 60.30, *p* < 0.0001; rimonabant administration is the main factor: F (1,16) = 0.3311, *p* = 0.5730; interaction of rimonabant administration × test condition: F (1,16) = 0.5003, *p* = 0.4895, Figure 3E] or inactive nose-pokes [test condition is the main factor: F (1,16) = 0.1657, *p* = 0.6894; rimonabant administration is the main factor: F (1,16) = 0.01475, *p* = 0.9048; interaction of rimonabant administration × test condition: F (1,16) = 0.002589, *p* = 0.9601, Figure 3F] between the two groups. In summary, rimonabant inhibits heroin-seeking behavior by blocking the CB1 receptor, but this process requires CS activation of heroin-associated memory.

### 3.4 Experiment 4: intraperitoneal injection of rimonabant 6 hours after memory reactivation did not attenuate heroin-seeking behavior

We studied whether rimonabant intervention outside of the reconsolidation window affects heroin seeking. During heroin self-administration training, no statistical difference was found in the number of active nose-pokes between the rimonabant group (n = 8) and the VEH group (n = 8) [training day is the main factor: F (9,126) = 9.761, *p* < 0.0001; rimonabant administration is the main factor: F (1,14) = 0.5697, *p* = 0.4629; interaction of rimonabant administration × training day: F (9,126) = 0.4735, *p* = 0.8901, Figure 4B]. Moreover, during the nose-poke extinction, no statistical difference was shown in the number of active nose-pokes between the two groups [extinction day is the main factor: F (9,126) = 65.83, *p* < 0.0001; rimonabant administration is the main factor: F (1,14) = 3.714, *p* = 0.0745; interaction of rimonabant administration × extinction day: F (9,126) = 0.2166, *p* = 0.9917, Figure 4C]. There was also no difference in the active nose-poke between the two groups in the reactivation test [different nose-pokes is the main factor: F (1,16) = 129.2, *p* < 0.0001; rimonabant administration is the main factor: F (1,16) = 0.0152, *p* = 0.9037; interaction of rimonabant administration × different nose-pokes: F (1,16) = 0.4883, *p* = 0.4961, Figure 4D].

The number of active and inactive nose-pokes did not differ between the two groups (*p* > 0.05) (Figure 4E) in the cue-induced reinstatement session. No appreciable difference was observed in the number of active nose-pokes between the rimonabant group and the control group in the heroin + cue-induced reinstatement test [test condition is the main factor: F (1,14) = 52.85, *p* < 0.0001; rimonabant administration is the main factor: F (1,14) = 0.4559, *p* = 0.5105; interaction of rimonabant administration × test condition: F (1,14) = 0.1855, *p* = 0.6732, Figure 4F]. No statistical difference was found between the two groups concerning the number of inactive nose-pokes [test condition is the main factor: F (1,14) = 0.8147, *p* = 0.3820; rimonabant administration is the main factor: F (1,14) = 0.004101, *p* = 0.9498; interaction of rimonabant administration × test condition: F (1,14) = 0.1381, *p* = 0.7157, Figure 4G]. These results demonstrated that rimonabant treatment outside of the window of memory reconsolidation did not weaken the heroin-seeking behavior.

## 4 Discussion

In this study, rimonabant treatment immediately after CS exposure significantly attenuated cue or heroin + cue-induced drug-seeking behavior and lasted for 28 days. Rimonabant administration without CS exposure or 6 hours after CS exposure had no such effect. These findings suggest the potential of rimonabant to disrupt memories associated with heroin use disorder during the process of memory reconsolidation. Rimonabant did not affect heroin seeking without CS exposure, implying that its impact on memory reconsolidation was contingent upon memory retrieval. Further, rimonabant impaired heroin-seeking behavior and maintained its effects over an extended period. These conclusions are in line with prior studies (Jian et al., 2014; Luo et al., 2015; Higginbotham et al., 2021b).

Drug memory can be modified when the memory becomes unstable before its reconsolidation. This process consists of the disruption of protein synthesis through inhibitors like anisomycin, rapamycin, sulfur dioxide, and berberine, which exhibit long-lasting effects on drug-seeking (Wu et al., 2011; Lin et al., 2014; Shen et al., 2020; Chen et al., 2021; Rulan et al., 2021; Zhang et al., 2021; Xie et al., 2022). Diergaarde L et al. found that propranolol injection after 10 min of CS exposure did not reduce sucrose-seeking behavior in sucrose self-administered rats, but it did after 20 min of cue exposure, suggesting that the duration of retrieval is critical (Diergaarde et al., 2006). The length of time spent in the operant context during reactivation is important. A retrieval session with insufficient time may not activate the memory trace and prevent destabilization. In our study, administering rimonabant 15 min after memory reactivation disrupted drug memories and reduced the likelihood of a subsequent reinstatement of heroin-seeking behavior, indicating that 15-min CS exposure is necessary for heroin-related memory reconsolidation (Qian et al., 2022).

Over time, drug memories can become stable again due to the resynthesis of proteins (Lee et al., 2017). Injecting rimonabant immediately after a 15-min CS exposure reduced subsequent heroin-seeking behavior. However, when rimonabant was administered 6 hours after CS exposure, it did not have an effect. These findings suggest that the disruptive effect of rimonabant depends on the instability of drug memory during reconsolidation window. Amnestic manipulations should be applied within a limited temporal period before the memory becomes restabilization.

We found that rimonabant when given immediately following CS exposure, decreased cue-induced and heroin + cue-induced drug-seeking behavior in rats; however, this effect was not observed when CS exposure was absent. This phenomenon suggests that CS exposure is crucial for rimonabant to interfere with heroin-associated memory. Additionally, rimonabant treatment had a prolonged inhibitory effect on heroin-seeking behavior, indicating that rimonabant disrupts the heroin-associated memory rather than temporarily inhibiting it. Preclinical and clinical trials have suggested that rimonabant may erase such a memory related to nicotine, cocaine, or fear (Ward et al., 2009; Fang et al., 2011; Lee et al., 2019). It is reported as a defining feature of memory that pharmacological manipulation after memory retrieval results in long-lasting amnesia subsequently (Nader and Hardt, 2009; Lee et al., 2017).

The impact of systematic rimonabant administration implies the involvement of CB1 receptors in memory reconsolidation. CB1 receptors are extensively dispersed and are highly dense in brain areas including the nucleus accumbens, hippocampus, basal ganglia, amygdala, prefrontal cortex, and cingulate gyrus (Hillard, 2014). The main location of CB1 receptors is the neuron terminal, where they can play the role of inhibiting neurotransmitter release. Activating CB1 receptors on GABA neurons in the ventral tegmental area (VTA) can lead to the inhibition of GABA release, thereby relieving its inhibition on dopaminergic neurons. CB1 receptor activation in the ventral tegmental area (VTA) may result in reduced glutamate release (Spanagel, 2020). Although the neural circuitry underlying heroin memory reconsolidation remains unclear, the amygdala may represent a central target within the circuit. For instance, Lee et al. found that infusion of rimonabant into the amygdala before CS exposure blocked the disruptive effects of MK-801 on reconsolidation (Lee et al., 2019). Glycogen synthase kinase 3 beta (GSK-3β) in the basolateral amygdala may function in the regulation of memory reconsolidation in heroin use disorder (Xie et al., 2022). CB1 receptors within the amygdala-prefrontal cortex pathway have been found to regulate neuronal plasticity and emotional memory encoding (Tan et al., 2010). Collectively, these findings suggest a pivotal role of the amygdala involved in heroin memory reconsolidation. Subsequent research endeavors may concentrate on the amygdala to delve further into the brain pathways by which CB1 receptors influence heroin memory reconsolidation. Notable that infusions of rimonabant in the amygdala before re-exposure and in the hippocampus after re-exposure impair the reconsolidation blocking of MK-801, possibly owing to the involvement of the CB1 receptor in memory instability caused by reactivation (Lee et al., 2019). Another study showed that rimonabant given before CS exposure did not interfere with the process of fear memory reconsolidation but blocked subsequent extinction, indicating that memory extinction is likewise mediated by CB1 receptors (Suzuki et al., 2004). According to their research, there may be distinct mechanisms involving memory instability that the CB1 receptor is engaged in. Studies have shown that rimonabant has a high affinity with mu-opioid receptors (MORs) and may exert inhibitory effects by directly binding to mu-opioid receptors (Seely et al., 2012). MORs play an important role in the hedonic and incentive reward reinforcement (Wang, 2019). The binding of MOR antagonists with MORs may weaken the reinforcing effects of heroin and the association between CS and opioids (O'Connor and Fiellin, 2000). This suggests that rimonabant’s disruption of heroin memory reconsolidation may not be solely related to CB1 receptors.

Several limitations should be addressed. Firstly, only male rats were used in our study. Male and female rats differ in their response to drugs in self-administration experiments due to the action of sex hormones. Females self-administer more opioids and stimulants than males do in rodents, as well as higher levels of drug-seeking during the processes of extinction and relapse (Lynch and Carroll, 2000; Ruda-Kucerova et al., 2015). However, drug background-induced cocaine-seeking behavior was attenuated independent of gender after reconsolidation (Ritchie et al., 2021). Therefore, the sex differences regarding the effect of rimonabant on reconsolidation remain for further evaluation. Secondly, the use of a novel context rather than a training box during CS exposure can avoid the influence of other factors. The investigation by employing a novel context is expected for comparison in the future study. Thirdly, rimonabant showed a prolonged effect on the reduction of heroin-seeking behavior, which may be partly due to the carry-over effects and extinction learning-altering responses at day 28. Lastly, rimonabant use has been linked to psychological side effects, including depression, suicidal thoughts, and gastrointestinal issues (Le Foll et al., 2009). Ettaro et al. revealed that no evidence of depressive or anxiety-like phenotypes was shown in male Sprague-Dawley rats at the dose of 3 mg/kg rimonabant (Ettaro et al., 2020). However, female Wistar rats developed anxiety and depression when treated with 3 mg/kg rimonabant during glucose withdrawal (Blasio et al., 2013). The distinct results of rimonabant may be possibly due to the difference of the species, sexes, or experiment procedures. Studies should be applied to clarify the effect of different doses of rimonabant on memory reconsolidation and the possible psychotoxic effects.

In conclusion, we employed an approach that combined self-administration protocols with retrieval experiments to investigate the impact of drug interventions on memory reconsolidation and subsequent heroin-seeking behavior. The results demonstrated that administering rimonabant during the critical time window following retrieval disrupts memory reconsolidation and reduces the likelihood of relapse. CS activation of heroin-associated memory is necessary for rimonabant to block the CB1 receptor and thus weaken the seeking behavior. These findings provide a therapeutic potential of rimonabant in the prevention of heroin relapse, which may shed new light on medication for substance use disorder.

## Data Availability

The raw data supporting the conclusion of this article will be made available by the authors, without undue reservation.
